# Transient dystonia correlates with parkinsonism after 1-methyl-4-phenyl-1,2,3,6- tetrahydropyridine in nonhuman primates

**DOI:** 10.3389/dyst.2023.11019

**Published:** 2023-02-01

**Authors:** S. A. Norris, L. Tian, E. L. Williams, J. S. Perlmutter

**Affiliations:** 1 Department of Neurology, School of Medicine, Washington University in St. Louis, St. Louis, MO, United States,; 2 Department of Radiology, School of Medicine, Washington University in St. Louis, St. Louis, MO, United States,; 3 Department of Neuroscience, School of Medicine, Washington University in St. Louis, St. Louis, MO, United States,; 4 Department of Physical Therapy, School of Medicine, Washington University in St. Louis, St. Louis, MO, United States,; 5 Department of Occupational Therapy, School of Medicine, Washington University in St. Louis, St. Louis, MO, United States

**Keywords:** striatum, MPTP, plasticity, monkey, substantia nigra

## Abstract

Unilateral internal carotid artery 1-methyl-4-phenyl-1,2,3,6-tetrahydropyridine (MPTP) infusion in non-human primates produces transient contralateral hemi-dystonia followed by stable contralateral hemi-parkinsonism; the relationship between dystonia and parkinsonism remains unclear. We hypothesized that transient dystonia severity following MPTP correlates with parkinsonism severity. In male Macaca nemestrina (*n* = 3) and M. fascicularis (*n* = 17) we administered unilateral intra-carotid MPTP, then correlated validated blinded ratings of transient peak dystonia and delayed parkinsonism. We also correlated dystonia severity with post-mortem measures of residual striatal dopamine and nigral neuron counts obtained a mean 53 ± 15 days following MPTP, after resolution of dystonia but during stable parkinsonism. Median latency to dystonia onset was 1 day, and peak severity 2.5 days after MPTP; total dystonia duration was 13.5 days. Parkinsonism peaked a median of 19.5 days after MPTP, remaining nearly constant thereafter. Peak dystonia severity highly correlated with parkinsonism severity (r[18] = 0.82, *p* < 0.001). Residual cell counts in lesioned nigra correlated linearly with peak dystonia scores (r[18] = −0.68, p=<0.001). Dystonia was not observed in monkeys without striatal dopamine depletion (*n* = 2); dystonia severity correlated with striatal dopamine depletion when residual nigral cell loss was less than 50% ([11] r = −0.83, *p* < 0.001) but spanned a broad range with near complete striatal dopamine depletion, when nigral cell loss was greater than 50%. Our data indicate that residual striatal dopamine may not reflect dystonia severity. We speculate on mechanisms of transient dystonia followed by parkinsonism that may be studied using this particular NHP MPTP model to better understand relationships of transient dystonia to nigrostriatal injury and parkinsonism.

## Introduction

The neurotoxin 1-methyl-4-phenyl-1,2,3,6-tetrahydropyridine (MPTP) produces relatively selective dopaminergic nigrostriatal injury (1). Systemic MPTP exposure in non-human primates (NHP) causes diffuse nigrostriatal injury followed by subsequent stable dopa-responsive parkinsonism, modeling motor manifestations of people with idiopathic Parkinson disease (2–5). Single dose intra-carotid (IC) administration in untreated baboons, *Macaque nemestrina*, and *Macaque fascicularis* can cause contralateral acute transient dystonia followed by parkinsonism (6, 7). Unilateral IC MPTP is advantageous, given the un-injected side sustains no changes to presynaptic nigrostriatal dopaminergic neurons (8, 9) and provides an internal control (e.g. pathologic comparison of the affected and unaffected brain hemispheres) for studies of pathophysiological mechanisms. To date, no study has characterized the relationship of dystonia and parkinsonism in NHPs following acute IC administration of MPTP.

A study of nigrostriatal reserve in dopaminergic cell bodies and their terminal fields following IC MPTP administration in NHPs demonstrated that parkinsonism ratings correlated with striatal dopamine but only when nigral dopamine cell loss did not exceed 50%. When nigral cell loss exceeds 50%, striatal dopamine levels reached nearly complete loss despite continued increased severity of parkinsonism with additional loss of nigral cell bodies of dopaminergic neurons (9). As a result, striatal PET measures of presynaptic nigrostriatal neurons may only be a valid biomarker of disease severity in only mild MPTP induced parkinsonism. The relationship between parkinsonism and acute MPTP-induced transient dystonia remains to be determined. Study of this relationship may provide insight regarding the pathophysiology of dystonia, particularly given experimental evidence linking both dystonia and parkinsonism to striatal dopaminergic dysfunction.

In this analysis of cross-sectional data, we determine novel relationships of transient dystonia following acute IC administration of MPTP to a) degree of subsequent parkinsonism, b) loss of tyrosine hydroxylase (TH) immunoreactive neuronal cell bodies in substantia nigra pars compacta, and c) loss of striatal dopamine. The current approach expands upon our prior reports of transient dystonia followed by parkinsonism (6), now specifically testing the hypothesis that the degree of transient dystonia after acute IC MPTP administration in non-human primates correlates with the severity of parkinsonism, and thus correlates with the degree of nigral damage. Our observation of relationships between transient dystonia, parkinsonism, nigral cell count, and residual striatal dopamine prompts discussion regarding potential mechanisms underlying transient dystonia followed by parkinsonism, and future promise for this model in dystonia related research.

## Materials and methods

### Subjects

#### Acute MPTP induced dystonia

We retrospectively studied 20 male macaque monkeys, 17 *Macaca fascicularis* and 3 *Macaca. nemestrina* before and after unilateral internal carotid artery infusion of various doses of MPTP; two additional monkeys were included as controls that did not receive MPTP. All animals included in the current study were selected from an existing cohort utilized for an experimental paradigm previously conducted to test hypotheses relating to pathophysiological mechanisms of parkinsonism. These parent biomarker studies relied on the minimum number of animals necessary to achieve statistical power for respective *a-priori* hypotheses. From this existing cohort, animals with severe acute MPTP toxicity (10) or exposure to dopaminergic medications or carboxyfullerene were excluded for the current analysis (11, 12). Furthermore, as the current study aimed to determine the relationship between peak acute transient dystonia and degree of parkinsonism, animals euthanized prior to anticipated peak parkinsonism were excluded. Two *nemestrina a*nimals had microelectrode penetrations in the globus pallidus on the infusion side before and after MPTP administration. Otherwise, no animals received intracranial manipulations during the experimental period. Animals were always able to care for themselves. The mean age of all animals was 7.3 ± 1.4 years (range, 4.9–9.6 years). Behavioral measures for a portion of these animals were previously reported (9), although blinded behavioral ratings of videos were repeated in the current study to maintain consistency across all animals since the prior blinded reviewer was no longer available. Data for all animals are presented in Table 1.

In accordance with the recommendations of the Weatherall report “The Use of Non-Human Primates in Research,” all steps were taken to ameliorate suffering in these studies. Guidelines prescribed by the NIH Guide for the Care and Use of Laboratory Animals (NIH Publications No. 8023, revised 1978) were followed in the parent biomarker studies, and all studies were approved by the institutional animal care and use committee at Washington University in St Louis.

### MPTP infusion

We infused the right internal carotid artery with MPTP in anesthetized animals using angiographic guidance as previously described (6). Briefly, animals fasted overnight and underwent anesthesia induction with ketamine and glycopyrrolate followed by intubation with a soft-cuffed endotracheal tube. Anesthesia was maintained with isoflurane inhalation. A 20-gauge catheter was placed into a femoral artery followed by fluoroscopic guidance of an 80-cm catheter guided into the right internal carotid artery over a guidewire. Cerebral angiogram prior to infusion confirmed proper placement of the catheter and good distal blood flow.

To obtain a dose response curve, a randomized dose between 0 and 0.25 mg/kg of MPTP (Sigma, St. Louis, MO) diluted in normal saline was infused IC in a concentration of 0.1 mg/mL at a rate no faster than 1 mL per minute. The mean absolute dose of MPTP administered was 1.28 mg ± 0.71 across monkeys injected (10). A repeat angiogram was performed following infusion of MPTP to document maintenance of proper positioning and distal blood flow. After the procedure, animals were continuously observed until able to care for themselves. Safety precautions were followed for handling MPTP and all contaminated tissues and waste products (13).

### Behavioral evaluations

Video was obtained at the same time of day (early morning) in the first 3 days following MPTP infusion, then several times per week after MPTP until euthanasia. The behavioral protocol included a) walking 15 feet toward, then away from the camera three times in a straight corridor, b) walking in a circle of approximately 7 feet diameter, 3 times clockwise then 3 times counterclockwise, and c) reaching 10 times with each hand for a food item held by the trainer at arm’s length from the animal. A single observer blinded to each animal’s treatment status rated each video clip, which was presented in a random order, using a scale previously developed and validated for non-human primate studies (7). Behavioral evaluations of parkinsonism and dystonia are consistent with those previously described (6, 7, 9).

Dystonic posturing was defined as extensor posturing of an upper or lower extremity and was clearly distinguished from the more typical parkinsonian flexed posture of the limb. Briefly, ratings for dystonia included a 0–3 scale (0 = unaffected, 1 = mildly affected, 2 = moderately affected, and 3 = severely affected) for dystonia occurring in each limb: 1) with the animal sitting at rest, 2) with movement of the rated limb(i.e., during gait or when reaching for a food item), and 3) movement of the contralateral limb. The ratings were scored for each side separately, producing a composite with a maximum dystonia rating of 18 per side. Ratings for parkinsonism included a 0–3 scale (0 = unaffected, 1 = mildly affected, 2 = moderately affected, and 3 = severely affected) for bradykinesia, tremor, and/or flexed posturing. The maximum score for parkinsonism ratings was also 18 per side. Behavioral raters were blind to *in vitro* measures (described below) and MPTP dose. Parkinsonism and dystonia ratings from some animals have been previously published (6, 9), but rating scores were reestablished to maintain rater consistency across all animals as the prior rater was no longer available to extend ratings to newly acquired animals.

### *In vitro* measures

Animals were euthanized with intravenous pentobarbital (100 mg/kg; Somnasol Euthanasia, Butler Schein Animal Health, Dublin, OH) at completion of the parent study. The brain was removed within 10 min. Details regarding animal euthanasia, brain retrieval and preparation, tyrosine hydroxylase immunohistochemistry and stereology, and striatal dopamine measurement protocols are described elsewhere (9), but we provide a brief overview here.

For substantia nigra tyrosine hydroxylase immunohistochemistry, the midbrain was fixed in 4% paraformaldehyde in 0.1 M phosphate buffered solution for 7 days, transferred to 30% sucrose for 7 days, and then cut on a freezing microtome in serial 50 μm-thick transverse sections after 7 days. Every 6th section was processed for tyrosine hydroxylase immunocytochemistry and sections were counterstained with cresyl violet for unbiased stereological counts of nigral neurons obtained *via* microscope digital image acquisition. Substantia nigra pars compacta was defined as the region ventral to medial lemniscus and lateral to the third cranial nerve fibers, and TH-stained nigral cells were counted using the Visiopharm Integrator System (version 3.2.10.0).

For striatal dopamine measures, separated hemispheres were sliced in the coronal plane at the time of euthanasia followed by collection of standard punch biopsies from caudate and putamen bilaterally which were frozen on dry ice snow and stored at −80°C until dopamine was assayed. High-performance liquid chromatography with electrochemical detection was applied to measure striatal dopamine levels, expressed in microgram per Gram of wet tissue.

All *in vitro* measurements were performed by a rater blinded to MPTP dose and behavioral measures. Select *in vitro* measures from animals included here have been published in-part elsewhere (9).

### Statistics

Mean, standard deviation, is reported for continuous variables of interest, while median is reported for ordinal data. Two-sample paired t-tests were used to compare continuous variables of interest between the MPTP-treated and control sides. We correlated non-parametric validated blinded ratings of transient peak dystonia scores with that of subsequent peak parkinsonism, *in vitro* measures of striatal dopamine, and unbiased stereologic counts of nigral neurons with TH by calculating a one-tailed Spearman’s coefficient (rho) in SPSS (IBM, version 22). Prior to data analysis, we conservatively defined the threshold of behavioral dystonia as a score of at least two points to avoid possible overlap with normal behavior, similar to our prior strategy with respect to parkinsonism (9).

## Results

Table 1 summarizes animal characteristics, behavioral, biochemical, and morphological profiles for all monkeys included in this study.

### Behavioral response to MPTP

**FIGURE 1:**
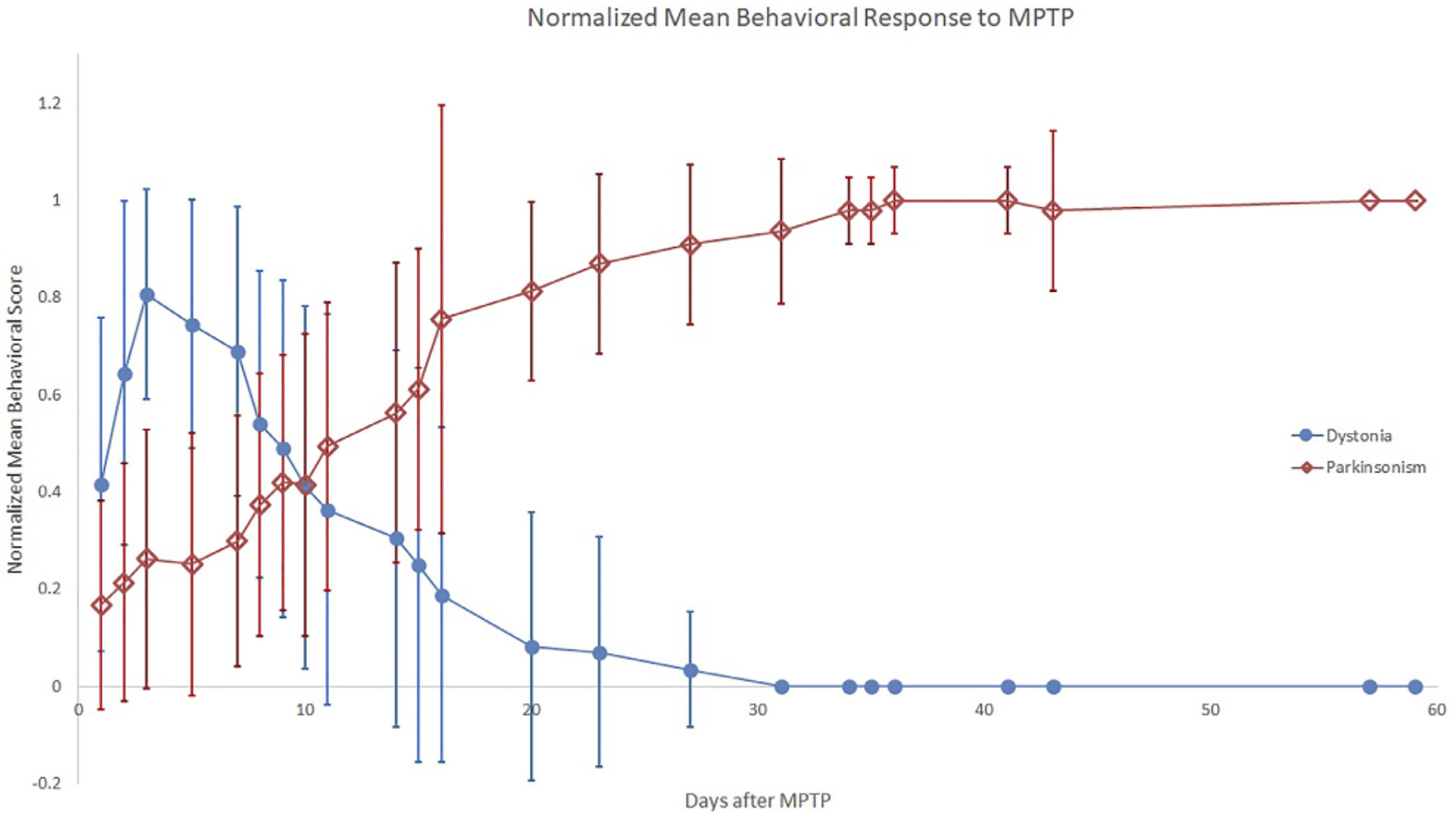
Normalized Mean Behavioral Response to MPTP. The plot displays mean ratios, across all monkeys per time bin, of individual dystonia (circle) or parkinsonism (diamond) scores divided by the individual monkey’s respective peak measure. Error bars represent standard deviation of the ratios across monkeys for each time bin.

The typical behavioral response to unilateral MPTP injection included recovery from anesthesia followed within hours to days by contralateral transient hemi-dystonia. Hemi-dystonia was observed in 16/18 MPTP injected monkeys as extension of the fore- and hind-limbs at the shoulder/hip, elbow/knee, and wrist/ankle with external rotation at the shoulder/hip that often worsened with attempted use of the affected limbs (either walking-worse when walking in circles with the affected side on the inner diameter- or reaching for objects). There was no postural tremor at onset of dystonic features in any of the observed monkeys. Four animals (two that did not receive MPTP) did not exhibit any dystonia or parkinsonism following MPTP administration, before euthanasia. The mean times following MPTP to onset of dystonic manifestations, peak dystonia and duration of dystonia are presented in Table 1. Hemi-dystonia gradually resolved after peak dystonia with overlapping onset of hemi-parkinsonism (Figure 1). At euthanasia, no animals had dystonic manifestations.

**FIGURE 2:**
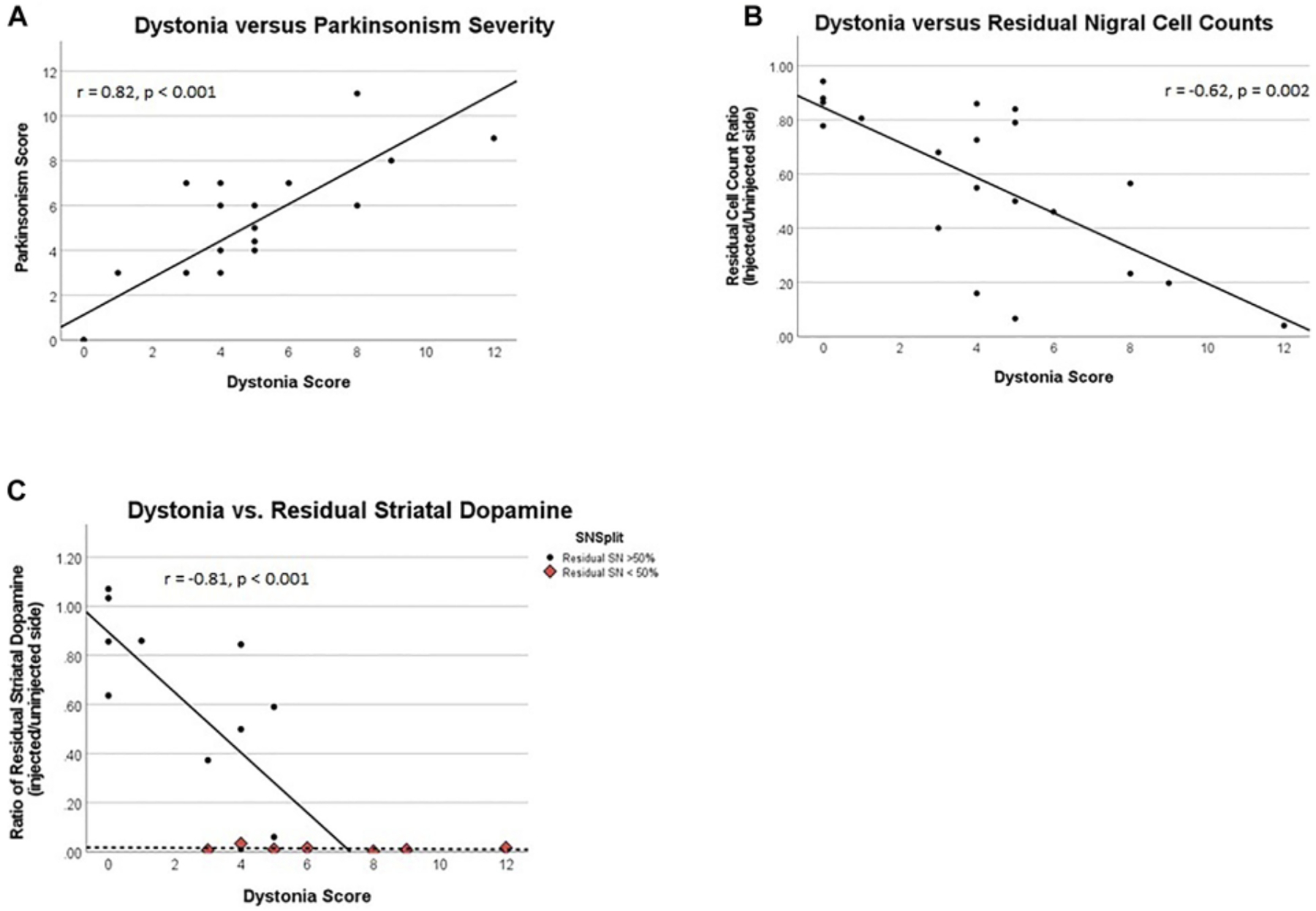
**(A)** Dystonia rating score vs. parkinsonism rating score, **(B)** Dystonia score vs. the ratio of residual substantia nigra cells, and **(C)** Dystonia vs. residual striatal dopamine. Correlation represents only data where residual SN > 50%, a distinct data cluster from that of SN < 50%. **(B, C):** All ratios represent their respective measures on the brain side injected with MPTP over similar measures for the un-injected side.

Peak dystonia score correlated with peak parkinsonism([18], r = 0.82, *p* < 0.001, Figure 2A). Mean time following MPTP to onset and plateau of parkinsonism at peak parkinsonism severity are presented in Table 1. The parkinsonian phase typically began with flexed posturing of the affected upper and lower extremity with bradykinesia. Some ambiguity existed with regard to scores of bradykinesia since severe dystonia often caused bradykinesia. In some animals, extensor dystonic posturing of the lower or upper extremity persisted when bradykinesia developed. Only two animals had tremor during the parkinsonian phase.

### Correlation of dystonia with delayed measures of nigral injury

Quantification of TH-stained nigral neurons was obtained a mean of 53 days following MPTP, in all cases acute transient dystonia resolved. The percent of residual nigral cell counts correlates with the prior peak dystonia rating scores ([18], r = −0.63, *p* = 0.002, Figure 2B). The threshold of nigral cell loss remains unknown given timing of TH-nigral measures in relation to dystonia, but monkey 6 represents the lowest threshold of injured nigra at 14% with associated dystonia, a similar level to that previously observed for parkinsonism (9).

### Correlation of dystonia with subsequent percent residual striatal dopamine

The peak dystonia rating score correlated linearly with the percent residual striatal dopamine obtained a mean of 53 days after MPTP (r = −0.81, *p* < 0.001), when nigral injury was less than 50%. When nigral injury exceeded 50%, the percent residual striatal dopamine was essentially zero across the entire measured range of dystonia scores. Data for residual striatal dopamine are displayed in Figure 2C; we only correlate data where nigral injury is less than 50% to avoid false representation of what appear as independent clusters. The threshold of striatal dopamine loss that corresponded to transient dystonia also remains unknown given timing of striatal dopamine measures in relation to dystonia, but the lowest threshold of striatal dopamine depletion was 14%–36% with associated prior transient dystonia, similar to that previously reported for parkinsonism (9). We averaged post-mortem striatal measures of dopamine for the caudate and putamen as the mean caudate dopamine concentrations did not differ from putamen measures on the control side (mean 11.9 ± 6.5 ug/g versus 12.1 ± 5.1 ug/g of wet tissue, respectively; two-sample paired t-test: t = −0.13, *p* = 0.90) and MPTP-lesioned side (5.4 ± 7.3 ug/g versus 5.4 ± 6.2 ug/g of wet tissue, respectively; two-sample paired t-test: t = −0.03, *p* = 0.97). Similarly, the mean caudate percent residual dopamine concentration did not differ from the putamen concentrations (41 ± 42% versus 43.7 ± 44%, respectively; two-sample paired t-test: t = −0.53, *p* = 0.61).

## Discussion

Our data build upon evidence that unilateral IC MPTP in non-human primates causes acute transient hemi-dystonia followed by stable parkinsonism, (6, 7) demonstrating that dystonia severity correlates with severity of parkinsonism. Our data further indicate that residual striatal dopamine does not reflect dystonia severity when nigral injury exceeds 50%, although measures of striatal depletion occurred after resolution of dystonia. Yet, dystonia severity strongly correlates with measures of dopaminergic nigral cell body injury, supporting a common mechanism underlying dystonia and parkinsonism that involves nigrostriatal injury (9). Interpretation of these data must be done with caution since the nigral measures were obtained following resolution of transient dystonia. Specifically, our results cannot be generalized to transient dystonia alone. Nevertheless, the relation of parkinsonism and nigral injury with transient dystonia offers clues that may guide future testable hypotheses aimed at understanding mechanisms of acute transient dystonia followed by parkinsonism in this non-human primate MPTP model.

### Relationship of transient dystonia and parkinsonism in the NHP MPTP model

Dystonia is not consistently reported before parkinsonism in primates treated with MPTP. Variability in MPTP primate models may contribute to this, including use of different ages of old- and new-world primates and variable administration and dosing methods that includes intracarotid, intramuscular, subcutaneous, or intravenous as a single dose or repeatedly over a few days, weeks, or months (14–22). Regardless, IC MPTP administration in baboons, *Macaca nemestrina*, and *Macaca fasicularis* has consistently caused transient dystonia followed by chronic parkinsonism (6, 7). It remains unclear whether differences in observed behavior results from methodology, or rather, lack of recognition and/or reporting of the early dystonic manifestation. The high correlation of dystonia and parkinsonian severity strengthens the association of MPTP with transient dystonia, strengthening support of this model for the study of pathophysiologic mechanisms and nigrostriatal function.

It is unlikely that subjective rating errors occurred when distinguishing phenomenology of dystonia and parkinsonism (i.e., mistaking early, more severe parkinsonism for dystonia) since the scores on these two rating scales clearly diverged as demonstrated in Figure 1. Also, a key rating observation helped to distinguish the dystonic from parkinsonian phase; we defined dystonia as extensor posturing of an extremity while flexion indicated parkinsonism.

### Striatal dopamine is an inadequate biomarker for transient dystonia

Animal studies have previously implicated the basal ganglia in dystonia [see reviews: (23–25)]. Selective striatal lesions with 6-hydroxydopamine or 3-nitropropionic acid and MPTP caused dystonia in rodents (26, 27) and non-human primates (6, 7, 28, 29). MPTP demonstrates selective preference to injure dopaminergic neurons (30–32). During the dystonic phase in baboons previously treated with IC MPTP, putaminal dopamine D2-like specific binding sites decreased about 30% (measured as soon as 10 days after MPTP) with associated striatal dopamine deficiency of about 97%–98% (33). In macaques, during the dystonic phase, striatal dopamine decreased about 66% in the first day (*n* = 2), 98% at day 3 (*n* = 1) and 99% at 2–4 months (*n* = 5) (6). Striatal FDOPA uptake decreased approximately 70%, a loss maintained 2–4 months following infusion (6). Thus, the dystonic phase begins, continues and ends during a period of sustained striatal dopamine deficiency. Based on our observations that transient dystonia occurs in context of incomplete, low intensity persistent striatal dopamine depletion is of particular interest regarding pathophysiology given that striatal healing is unlikely. Our findings indicate that striatal dopamine alone does not reflect dystonia severity in this model through the full range of severity.

*Macaque nemestrina* and *Macaque fascicularis* animals that develop dystonia following IC MPTP (0.1–0.4 mg/kg) have quicker drops in striatal dopamine during the dystonic phase (6) than squirrel monkeys systemically administered MPTP for which dystonia was not reported (34). The squirrel monkeys have only modest drops in putamen dopamine at day 1 following subcutaneous administration of 2.5 mg/kg followed by further decreases at day 5 in both caudate and putamen. Thus, the rapid changes induced by IC MPTP that is completely extracted on first pass through the brain circulation may be critical to induce dystonia, above and beyond striatal dopamine deficiency alone.

### Correlations of nigral injury and dystonia severity warrants cautious interpretation

The temporal dissociation of transient dystonia and biochemical measures limits our ability to speculate regarding the correlation of dystonia severity with residual nigral cell counts. This association may reflect changes in the effects of dopamine transmission and/or receptor configuration in downstream striatal terminals, for which future studies are required to understand the timing of nigral injury in relation to transient dystonia.

### Potential for future applications of the NHP MPTP dystonia model

A critical question in our non-human primate IC MPTP model relates to what causes dystonia to recover whereas parkinsonism does not. Alterations in striatal dopaminergic signaling relate to the pathophysiology of both dystonia and parkinsonism (35, 36). Reports regarding the effects of MPTP on dopamine receptors vary. In general, there appears relatively little change in D1-like receptor numbers. Some report decreased dopamine D2-like receptors, but others report no change or an increase (37–41). As multiple dopamine receptors are the products of separate genes (42), it is possible that changes in D2-like receptor binding described in prior studies may be attributable to changes in the expression of a single receptor subtype or to complex changes in the expression of D2, D3, or D4 receptors. Future studies focused on potentially adaptive relationships of various dopamine receptors during the transition from dystonia to parkinsonism, particularly differentiating the direct and indirect pathway, may be valuable to understand the role of such receptors in altered neural plasticity and phenotypic conversion from dystonia to parkinsonism. Changes in other modulators of the direct and indirect pathways such as such as dynorphin, enkephalin, substance P and PDE10a may also play a role in development of dystonia, and future molecular studies measuring their levels in this NHP model during and after transient dystonia may prove informative regarding pathophysiologic mechanisms of transient dystonia.

Acute transient dystonia in the current NHP model may also relate to abnormal cortico-striatal neural plasticity (43). In rodents, MPTP induced nigro-striatal injury produces immediate striatal release of dopamine and its metabolites (44, 45). Early profound dopaminergic efflux likely stimulates D1 and D2-like dopamine receptors simultaneously, possibly causing acute alterations in glutamatergic sensitivity *via* altered synaptic long term potentiation and long term depression at medium spiny neurons (46). Thus, IC MPTP may cause a sudden flood of available dopamine followed by marked reduction (6). This rapid dopaminergic striatal efflux followed by relative dopaminergic paucity mimics the clinical scenario of human cocaine exposure (i.e., striatal dopaminergic priming), where those exposed are more prone to drug-induced dystonia following dopamine receptor blockade (47). Acute dopamine depletion may produce more excitable medium-sized spiny neurons, less excitable GABAergic interneurons and increased cholinergic cell excitability (48, 49); thus, dopamine depleted striatal circuits exhibit pathological hyperactivity. Longitudinal neurophysiological, PET, and functional neuroimaging experiments flanking development and resolution of transient dystonia in this NHP MPTP model may unravel potential links between biochemical and circuit-level dysfunction contributing to altered cortico-striatal plasticity.

Each of these potentially contributing mechanisms of altered striatal plasticity requires future study, but the most intriguing relates to cholinergic interneurons (ChI). ChIs show hyperactivity with bursts and silences in the dopamine depleted striatum, where hyperactivity also relates to extrinsic synaptic inputs targeting ChIs *via* glutaminergic and GABAergic inputs. The latter is essential for sustaining ChI hyperactivity, but MPTP induced dopamine loss alters substantia nigra pars compacta signaling which reduces glutamate co-excitation of dorsolateral striatal ChIs due to downregulation of mGluR1 (50). As the relative ratio of striatal acetylcholine/dopamine relates to acute dystonia in mice treated with acute reserpine, one might anticipate that this ratio would normalize with downregulation of mGluR1, potentially translating to the end of the dystonic phase. This speculation supports growing data that ChIs play a role in dystonia and may serve as the main drivers of pathological hyperactivity in the striatum secondary to dopamine depletion and altered extrinsic synaptic inputs (51). Post-mortem histopathological measures of choline acetyltransferase (ChAT) or *in vivo* PET measures of [18F]fluoroethoxybenzovesamicol, (−)-[18F] FEOBV,(−)-(2R,3R)-trans-2-hydroxy-3-(4- phenylpiperidino)-5-(2-[18F]fluoroethoxy)-1,2,3,4-tetralin (52) or (−)-(1-(8-(2-[(18)F)fluoroethoxy)-3-hydroxy-1,2,3,4-tetrahydronaphtalen-2-yl)-pi peridi n -4 -yl) (4-fluorophenyl)methanone([18F]VAT) (53) may provide clues with respect to altered ChI activity during the transient dystonia phase in this NHP MPTP model.

Future studies are critical to tease out mechanistic components in the NHP model of transient dystonia which may include shifting neurotransmitter levels, immunomodulatory effects secondary to cellular injury, direct nigrostriatal influence versus indirect downstream effects, receptor auto regulation, altered transcription pathways, or shifts in network-level synchronicity.

### Synaptic sprouting does not explain phenomenological evolution

Given the acute nature of IC MPTP in our animals, one might speculate that initial terminal field injury causes dystonia with recovery of function (i.e. sprouting of terminal fields), relieving dystonia. However, there is no evidence in our animals to support sprouting of terminal fields that produces any functional activity or neuronal recovery during the dystonic phase or beyond (54).

## Conclusion

Severity of transient IC MPTP induced dystonia correlates with subsequent parkinsonism severity, supporting common pathways with respect to mechanistic consideration. The degree of dystonia correlates with subsequent nigral cell injury across the entire spectrum of severity, where striatal dopamine measures do not. There are limitations to interpreting the nigral cell counts, but sustained striatal dopamine depletion is not sufficient as a biomarker for transient dystonia. Although the dystonic phase is discrete and short lived in *Macaca fasiculata* and *Macaca nemestrina*, it is consistent and sufficient in duration to permit analysis of behavioral response in relation to physiological parameters, particularly with regard to studying dystonia independent of, or in relation to parkinsonism in unmedicated animals.

## Figures and Tables

**TABLE 1:** Animal characteristics and outcome measures.

Monkey	Species	Sex	Age (yrs)	Animal	MPTP dose	MPTP dose	Euthanasia	Parkinsonism	Parkinsonism	Parkinsonism	Dystonia	Dystonia	Dystonia	Dystonia	SN TH	Striatal DA
			At MPTP	Weight (kg)	(mg/kg)	(mg)	(days)	Peak rating	Onset (days)	Plateau (days)	Peak rating	Onset (days)	Peak (Days)	Duration (days)	(inj/uninject)	(inj/uninject)
1	Nemestrina	M	5.1	5.1	0.12	0.61	56	3	13	20	4	1	3	12	0.73	0.50
2	Nemestrina	M	5.6	5.6	0.06	0.34	56	3	16	20	1	1	1	13	0.81	0.86
3	Nemestrina	M	7.8	7.8	0.18	1.41	56	7	4	28	3	1	5	20	0.40	0.01
4	Fasicularis	M	6.5	6.45	0	0	56	0	N/A	N/A	0	N/A	N/A	N/A	0.78	1.07
5	Fasicularis	M	7.5	7.5	0.25	1.88	58	6	2	21	4	3	9	14	0.16	0.03
6	Fasicularis	M	5.6	5.6	0.12	0.672	56	4	9	11	4	1	1	5	0.86	0.84
7	Fasicularis	M	8.9	8.9	0.18	1.6	60	6	2	14	5	1	2	9	0.84	0.06
8	Fasicularis	M	7.0	6.95	0.06	0.417	56	3	8	18	3	1	3	10	0.68	0.37
9	Fasicularis	M	7.3	7.3	0.25	1.825	56	7	16	24	6	1	2	17	0.46	0.02
10	Fasicularis	M	7.0	7	0	0	57	0	N/A	N/A	0	N/A	N/A	N/A	0.88	1.03
11	Fasicularis	M	7.5	7.45	0.14	1.043	56	4	1	15	5	1	3	9	0.79	0.59
12	Fasicularis	M	8.2	8.2	0.14	1.148	57	9	1	24	12	1	1	7	0.06	0.02
13	Fasicularis	M	6.9	6.9	0.11	0.69	60	5	1	23	5	1	3	14	0.50	0.01
14	Fasicularis	M	8.9	8.9	0.14	1.25	57	8	1	18	9	2	4	15	0.20	0.01
15	Fasicularis	M	5.7	5.7	0.12	0.684	57	0	N/A	N/A	0	N/A	N/A	N/A	0.87	0.64
16	Fasicularis	M	4.9	4.9	0.06	0.294	57	0	N/A	N/A	0	N/A	N/A	N/A	0.94	0.86
17	Fasicularis	M	9.5	9.5	0.25	2.38	22	11	1	17	8	1	2	15	0.57	0.01
18	Fasicularis	M	9.0	9	0.25	2.25	21	7	6	20	4	1	1	15	0.55	0.01
19	Fasicularis	M	8.4	8.35	0.25	2.09	25	6	8	16	8	1	3	16	0.23	0.00
20	Fasicularis	M	9.6	9.55	0.25	2.39	87	4	5	19	5	1	2	8	0.07	0.01
Mean/Median^†^			7.3	7.33	0.16^a^	1.28^a^	53.3	5.5^†a^	4.5^†^	19.5^†^	4.5^†a^	1^†^	2.5^†^	13.5^†^	0.57	0.35
Standard Deviation			1.4	1.45	0.07^a^	0.71^a^	14.9								0.42	0.41

a Does not include 2 animals that did not receive MPTP.

† denotes median values; all others in the row are mean values.

SN TH, substantia nigra tyrosine hydroxylase; DA, dopamine; inj/uninjected = ratio of the left (MPTP injected) to right (uninjected) sides.

## Data Availability

Animal videos will only be released upon institutional approval to researchers qualified and trained in conjunction with an appropriate institutional animal care and use committee. Requests to access the datasets should be directed to JP, perlmutterjoel@wustl.edu.
